# Supplementary material for: Doubly Robust Estimation of Marginal Cumulative Incidence Curves for Competing Risk Analysis
Source: Stat Med. 2025 Aug 8;44(18-19):e70066. doi: 10.1002/sim.70066 (PMC12333911; doi:10.1002/sim.70066)
Supplement: Supplementary file 1 — Data S1. Supporting Information. [file SIM-44-0-s001.pdf]

Supplementary Section S1. True cumulative incidence curve

To formulate the true treatment specific conditional cumulative incidence curve for the cause-specific hazard functions $\lambda_k^z(x_1,x_2)$ described in $Section$ 3.2 of the main text, we define the all-cause hazard as $\lambda^z(x_1,x_2) = \sum_{k=1}^{K} \lambda_k^z(x_1,x_2)$, and the all-cause survival function as $S^z(t,x_1,x_2) = 1 - \exp(-t\,\lambda^z(x_1,x_2))$.[1] The true conditional cumulative incidence curve can be calculated as:

$$I_k^z(t,x_1,x_2) = \frac{\lambda_k^z(x_1,x_2)}{\lambda^z(x_1,x_2)} S^z(t,x_1,x_2)$$

To compute the treatment specific marginal cumulative incidence curve, we integrate over the distributions of X_1 and X_2 as follows:

$$I_k^z(t) = \int_{-\infty}^{\infty}\int_{-\infty}^{\infty} I_k^z(t,x_1,x_2) f_{x_1}(x_1) f_{x_2}(x_2)\, dx_1\, dx_2$$

With $f_{x_1}(x_1)$ and $f_{x_2}(x_2)$ denoting the density functions of covariates X_1 and X_2. In Scenario 1 and Scenario 3, we integrate over the covariate distribution of the total set of observations from both treatment groups. In Scenario 2 and Scenario 4, the control group $Z = 0$ represents an unbiased sample from the population of interest, hence we integrate only over the covariate distribution of the control group $Z = 0$. Lastly, since the discrete covariate X_1 can only take 3 values, each with 1/3 probability, we performed the computations in R by averaging over the 3 values of X_1 rather than to integrate over the categorical distribution:

$$I_k^z(t) = E_{X_1}\left[\int_{-\infty}^{\infty} I_k^z(t,x_1,x_2) f_{x_2}(x_2)\, dx_2\right]$$

The integral of the continuous covariate was obtained numerically using the R function integrate().[2]

Supplementary Section S2. Violation of the positivity assumption

An additional simulation was performed to examine the effect of a violated positivity assumption on the performance of the covariate adjustment methods. This simulation uses the settings from Scenario 1, as described in *Section* 3.1 of the main text. Instead of using $\omega = (1, -1, 1)$, we used $\omega = (6, -1, 1)$ as the coefficients for the logistic model for treatment assignment. This results in a drastic increase in the allocation of observations with $X_1 = 1$, (and $X_2 = 0$ since X_1 and X_2 encode indicator variables for three categories) to the treatment group ($Z = 1$). In doing so, $P(Z = 0|X_1 = 1, X_2 = 0)$ is close to 0, leading a to very small number of observations with $X_1 = 1$ in the control group, which is shown in supplementary Figure S1A. This results in propensity scores close to zero in the control group and close to one in the treatment group, indicating a violation of the positivity assumption (supplementary Figure S1B). We observe that this mostly adversely affects the performance of the IPW and doubly robust methods, that use propensity scores, leading to high variance and increased RMSE especially for estimation of curves under the control condition (see supplementary Figure S1C and supplementary Table S1). In comparison, the results from outcome regression remain unbiased and stable. Note that this is to be expected as the outcome model only has main effects of covariates and treatment (and no interaction between them). For each of these covariates there is sufficient data to estimate their main effect in the outcome model.

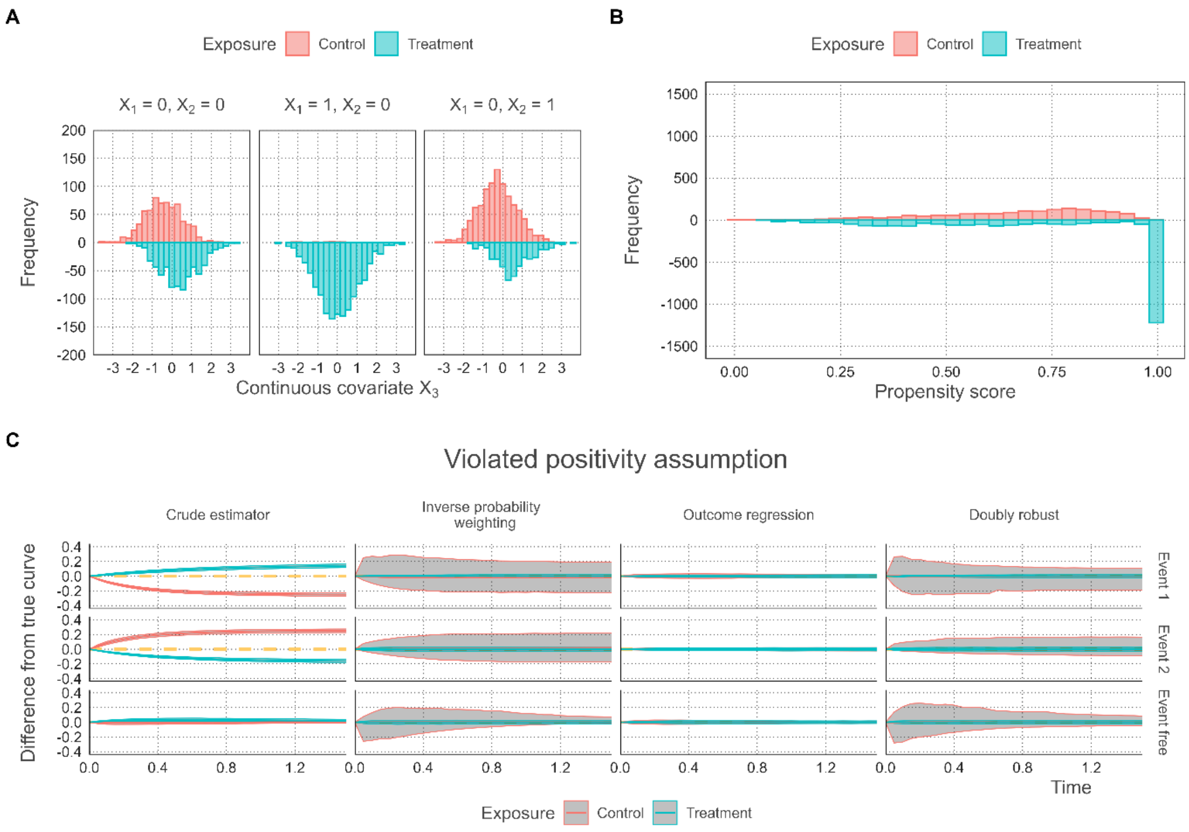

Figure S1. (A) Frequency distributions, stratified by treatment and covariates (B) The distribution of propensity scores in the two treatment groups. (C) The results from the simulation study with $M = 1000$ iterations. Mean differences between the true cumulative incidence of event 1, 2, and event free survival, and the estimated results using the naïve estimator (no covariate adjustment), inverse probability weighted estimator, outcome regression estimator, and doubly robust estimator in the two treatment groups. The bands indicate the 2.5th and 97.5th percentiles.

Table S1. Simulation results under violation of the positivity assumption, at approximately halfway the follow up ($t = 0.8$). Bias and root-mean squared error (RMSE) of the naïve estimator (no covariate adjustment), inverse probability weighted estimator, outcome regression estimator, and doubly robust estimator of the cumulative incidence are given for the cumulative incidence of event 1 and event 2, and event free survival.

**S1. Violated positivity (t = 0.8)**		**CI event 1**		**CI event 2**		**Event free survival**	
		**Bias**	**RMSE**	**Bias**	**RMSE**	**Bias**	**RMSE**
**Crude estimator**	**Control**	-0.23439	0.23463	0.23682	0.23715	-0.00243	0.00932
	**Treatment**	0.10869	0.10921	-0.13926	0.13958	0.03057	0.03244
**Inverse probability**	**Control**	-0.02228	0.11614	0.01648	0.09392	0.00579	0.05497
	**Treatment**	0.00022	0.00994	-0.00085	0.01458	0.00062	0.01283
**Outcome regression**	**Control**	0.00044	0.01439	-0.00019	0.01100	-0.00025	0.00961
	**Treatment**	0.00026	0.00830	-0.00062	0.01086	0.00036	0.01067
**Doubly robust**	**Control**	-0.00237	0.08562	0.00038	0.06317	0.00198	0.06069
	**Treatment**	0.00012	0.00960	-0.00064	0.01310	0.00052	0.01235

Supplementary Section S3. Data collection and classification

The Netherlands Cancer Registry (NCR), which is hosted by the Netherlands Comprehensive Cancer Organisation (IKNL), consists of data on all malignancies diagnosed in the Netherlands from 1989 onwards. Trained data managers obtain data on patient-, tumour- and treatment-related characteristics directly from patient files. Individual records were pseudonymized prior to data delivery. All women included in this study received a diagnosis of primary invasive breast cancer in 2005 (stage T1-2N0-1M0). Patients were treated in a hospital in the Netherlands, during which they underwent either mastectomy or breast-conserving surgery with adjuvant radiation therapy as the treatment of interest. Patient-, tumour-, treatment and hospital-related characteristics have been summarized in supplementary Table S1. Characteristics related to tumour staging were based on the pathological tumour characteristics, and described in accordance with the 6th edition of the tumour size, node, and metastasis classification system (TMN) for breast cancer.[3] Tumour grade, topology, and morphology were classified based on the 3rd edition of the International Classification of Diseases for Oncology.[4] The patients' socioeconomic status (SES) was determined at the time of diagnosis, and obtained per zip code area from the publicly available socioeconomic status records of The Netherlands Institute for Social Research.[5] Over a period of 10 years, patients were followed until one of three clinical outcomes occurred, or until they were censored from the study. From these patients, 5147 patients (61.0 %) were treated by breast-conserving therapy, and 3462 patients (39.0 %) were treated by mastectomy.

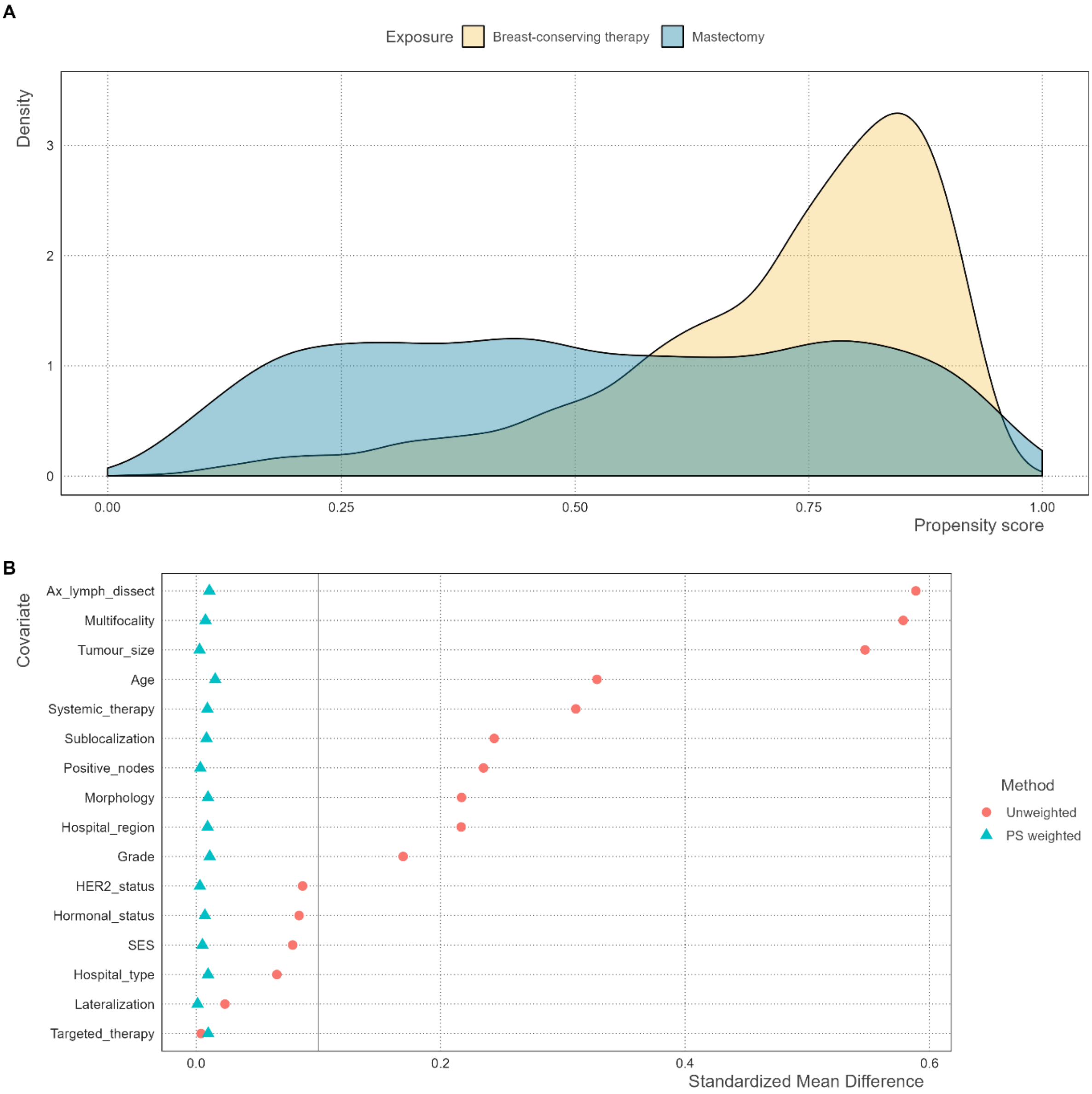

Figure S2. (A) Density distribution of the propensity scores for Mastectomy, plotted separately for patients who received breast-conserving therapy (yellow; light) and for those who received mastectomy (blue; dark). The propensity scores were obtained from a logistic regression of treatment as a function of relevant patient-, tumour- , treatment- and hospital-related covariates, after which the predicted response values were extracted from the model. (B) Love plot indicating the standardized mean differences of unweighted and propensity score weighted covariates.

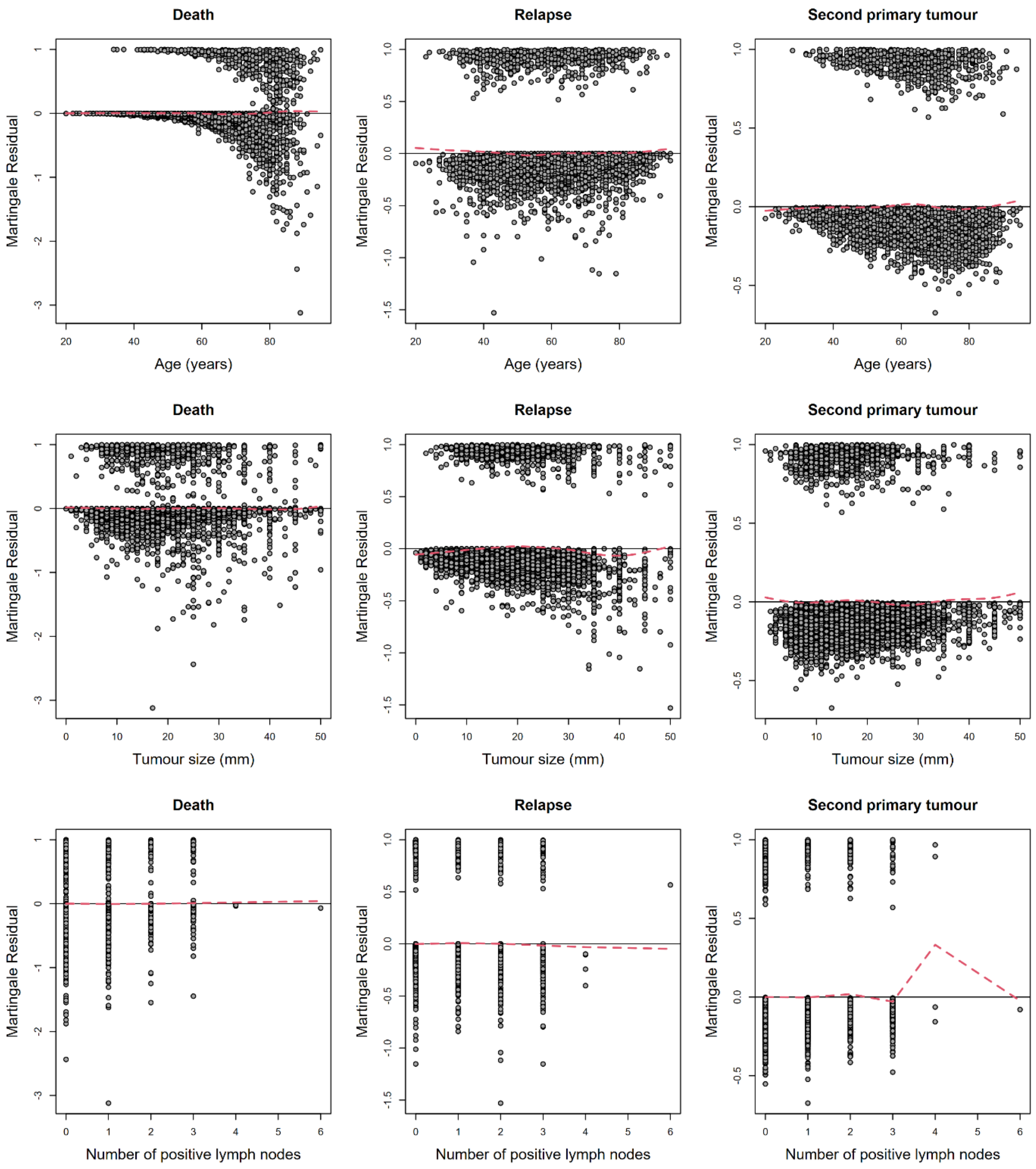

Figure S3. Assessment of the linearity assumption used in the cause-specific Cox regression models was performed by plotting Martingale residuals from these models against the three continuous predictors. The red dotted line represents a smooth spline between the Martingale Residuals and continuous predictor (df=7). Based on visual inspection, we did not find considerable signs of deviation from linearity for the three continuous covariates.

Table S2. Distribution of tumour-, patient-, treatment-, and hospital-related characteristics stratified by mastectomy and breast-conserving therapy. P values were obtained using χ^2 tests from logistic regressions with treatment allocation as outcome. Differences in the distribution of covariates between treatment groups was considered statistically significant at $p < 0.05$.

Characteristics	Mastectomy (*n* = 3462)		Breast-conserving therapy (*n* = 5417)		*p* value
Age (years)					
< 40	221	(6.4)	328	(6.1)	**< 0.001**
40-49	534	(15.4)	978	(18.0)	
50-59	783	(22.6)	1690	(31.2)	
60-69	690	(19.9)	1506	(27.8)	
70-79	694	(20.1)	787	(14.5)	
>= 80	540	(15.6)	128	(2.4)	
Mean tumour size in mm (IQR)	20	(13-25)	16	(11-20)	**< 0.001**
Mean number of positive lymph nodes (IQR)	1	(0-1)	0	(0-0)	**< 0.001**
Grade					
1	655	(18.9)	1460	(26.9)	0.088
2	1557	(45.0)	2340	(43.2)	
3	1037	(30.0)	1407	(26.0)	
Unknown	213	(6.1)	210	(3.9)	
Multifocality					
No	2480	(71.6)	4963	(91.6)	**< 0.001**
Yes	881	(25.5)	343	(6.3)	
Unknown	101	(2.9)	111	(2.1)	
Histological tumour type					
Ductal	2664	(76.9)	4513	(83.3)	**< 0.001**
Lobular	483	(14.0)	448	(8.3)	
Mixed	182	(5.3)	174	(3.2)	
Other	133	(3.8)	282	(5.2)	
Sublocalisation					
Outer quadrants	1525	(44.0)	2717	(50.2)	**< 0.001**
Inner quadrants	581	(16.8)	1164	(21.5)	
Central parts	318	(9.2)	316	(5.8)	
Overlapping lesions	949	(27.4)	1125	(20.8)	
Unknown	89	(2.6)	95	(1.7)	
Lateralisation					
Left	1777	(51.3)	2810	(51.9)	0.080
Right	1685	(48.7)	2607	(48.1)	
Hormonal receptor status					
Negative	532	(15.4)	803	(14.8)	0.080
Mixed	593	(17.1)	837	(15.5)	
Positive	2067	(59.7)	3458	(63.8)	
Unknown	270	(7.8)	319	(5.9)	

Characteristics	Mastectomy (*n* = 3462)		Breast-conserving therapy (*n* = 5417)		*p* value
HER2 status					
Negative	2318	(66.9)	3875	(71.5)	0.095
Unclear	196	(5.7)	297	(5.5)	
Positive	454	(13.1)	597	(11.0)	
Unknown	494	(14.3)	648	(12.0)	
Targeted therapy (trastuzumab)					
No	3302	(95.4)	5170	(95.4)	0.307
Yes	160	(4.6)	247	(4.6)	
Adjuvant systemtic therapy					
None	1463	(42.3)	2978	(55.0)	0.097
Chemotherapy	305	(8.8)	555	(10.2)	
Endocrine therapy	965	(27.9)	879	(16.2)	
Both	729	(21.0)	1005	(18.6)	
Axillary lymph node dissection					
No	1501	(43.4)	3809	(70.3)	**< 0.001**
Yes	1961	(56.6)	1608	(29.7)	
Socioeconomic status					
Low	1052	(30.4)	1482	(27.4)	0.051
Medium	1389	(40.1)	2141	(39.5)	
High	1021	(29.5)	1794	(33.1)	
Hospital type					
General	1407	(40.7)	1977	(36.5)	**< 0.01**
Top-clinical	1742	(50.3)	2952	(54.5)	
Academic	313	(9.0)	488	(9.0)	
Hospital region					
A	578	(16.7)	1078	(19.9)	**< 0.001**
B	218	(6.3)	320	(5.9)	
C	203	(5.8)	409	(7.6)	
D	436	(12.6)	793	(14.6)	
E	370	(10.7)	487	(9.0)	
F	634	(18.3)	631	(11.6)	
G	255	(7.4)	355	(6.6)	
H	345	(10.0)	556	(10.3)	
I	423	(12.2)	788	(14.5)	

Numbers in brackets indicate the percentage (%) of observations that correspond to the subgroup, *IQR* denotes the interquartile range for continuous covariates

Table S3. Coefficient table of the propensity score model estimated using logistic regression. Treatment allocation (Breast-conserving therapy = 0, Mastectomy = 1) was modeled as a function of tumour-, patient-, treatment-, and hospital-related characteristics. The estimates and their standard errors (SE) are reported on the log odds (logit) scale.

	Estimate	SE	*z*-value	Pr(>|*z*|)
(Intercept)	-4.492	0.264	-17.01	**< 0.001**
Age (years)	0.032	0.003	10.68	**< 0.001**
Tumour size in mm	0.067	0.004	16.13	**< 0.001**
Number of positive lymph nodes	-0.195	0.049	-3.98	**< 0.001**
Grade (1 = reference)				
2	0.066	0.079	0.84	0.40
3	0.158	0.098	1.60	0.11
Multifocality (No = reference)				
Yes	1.789	0.091	19.62	**< 0.001**
Histological tumour type (Ductal = reference)				
Lobular	0.313	0.101	3.08	**< 0.001**
Mixed	0.405	0.148	2.73	**< 0.01**
Other	-0.179	0.154	-1.16	0.25
Sublocalisation (Outer quadrants = reference)				
Inner quadrants	0.021	0.079	0.26	0.79
Central parts	0.606	0.113	5.37	**< 0.001**
Overlapping lesions	0.276	0.074	3.74	**< 0.001**
Lateralisation (Left = reference)				
Right	0.072	0.059	1.21	0.22
Hormonal receptor status (Negative = reference)				
Mixed	0.124	0.137	0.91	0.37
Positive	-0.045	0.127	-0.35	0.73
HER2 status (Negative = 0)				
Mixed	-0.015	0.120	-0.13	0.90
Positive	0.301	0.108	2.80	**<0.01**
Targeted therapy (No = reference)				
Yes	-0.218	0.163	-1.34	0.18

	Estimate	SE	*z*-value	Pr(>|*z*|)
Adjuvant systemic therapy (None = reference)				
Chemotherapy	-0.175	0.151	-1.16	0.25
Endocrine therapy	-0.219	0.092	-2.39	**< 0.05**
Both	-0.090	0.111	-0.81	0.42
Auxillary lymph node dissection (No = reference)				
Yes	1.133	0.076	14.89	**< 0.001**
Socioeconomic status (Low = reference)				
Medium	-0.092	0.078	-1.18	0.24
High	0.016	0.072	0.23	0.82
Hospital type (General = reference)				
Top-clinical	-0.169	0.067	-2.54	**< 0.05**
Academic	0.006	0.117	0.05	0.96
Hospital region (A = reference)				
B	0.303	0.128	2.37	**< 0.05**
C	-0.147	0.134	-1.10	0.27
D	-0.159	0.110	-1.45	0.15
E	0.148	0.121	1.22	0.22
F	0.467	0.107	4.36	**< 0.001**
G	0.287	0.128	2.25	**< 0.05**
H	0.111	0.113	0.99	0.32
I	-0.002	0.110	-0.02	0.99

Table S4. Coefficient table following cause-specific hazard modeling of death, relapse, and secondary tumour development. Cause-specific log hazard ratios (estimate), hazard ratios (HR), and standard errors (SE) are given for each tumour-, patient-, treatment-, and hospital-related covariate.

Death	Estimate	HR	SE	*z*-value	Pr(>|*z*|)
Treatment (Breast-conserving therapy = reference)					
Mastectomy	0.572	1.772	0.097	5.92	**< 0.001**
Age (years)	0.105	1.110	0.005	19.10	**< 0.001**
Tumour size in mm	0.017	1.017	0.005	3.51	**< 0.001**
Number of positive lymph nodes	0.069	1.071	0.065	1.04	0.30
Grade (1 = reference)					
2	0.006	1.006	0.102	0.06	0.95
3	-0.043	0.958	0.132	-0.32	0.75
Multifocality (No = reference)					
Yes	-0.003	0.997	0.124	-0.03	0.98
Histological tumour type (Ductal = reference)					
Lobular	-0.283	0.754	0.138	-2.02	**< 0.05**
Mixed	0.127	1.136	0.200	0.70	0.49
Other	0.201	1.223	0.174	1.17	0.24
Sublocalisation (Outer quadrants = reference)					
Inner quadrants	-0.127	0.881	0.112	-1.08	0.28
Central parts	-0.246	0.782	0.147	-1.75	0.08
Overlapping lesions	-0.041	0.959	0.099	-0.43	0.67
Lateralisation (Left = reference)					
Right	-0.027	0.973	0.080	-0.34	0.74
Hormonal receptor status (Negative = reference)					
Mixed	0.055	1.057	0.173	0.31	0.76
Positive	0.015	1.015	0.156	0.09	0.93
HER2 status (Negative = reference)					
Mixed	0.001	1.001	0.154	0.01	0.99
Positive	-0.169	0.845	0.146	-1.17	0.24
Targeted therapy (No = reference)					
Yes	-0.906	0.404	0.484	-1.85	0.06

Death	Estimate	HR	SE	*z*-value	Pr(>|*z*|)
Adjuvant systemic therapy (None = reference)					
Chemotherapy	0.312	1.366	0.224	1.30	0.19
Endocrine therapy	-0.229	0.796	0.108	-2.05	**< 0.05**
Both	-0.363	0.696	0.237	-1.55	0.12
Auxillary lymph node dissection (No = reference)					
Yes	0.294	1.342	0.097	2.97	**< 0.001**
Socioeconomic status (Low = reference)					
Medium	-0.057	0.945	0.094	-0.60	0.55
High	-0.182	0.834	0.104	-1.76	0.08
Hospital type (General = reference)					
Top-clinical	-0.062	0.940	0.088	-0.69	0.49
Academic	0.120	1.128	0.178	0.67	0.51
Hospital region (A = reference)					
B	0.188	1.207	0.170	1.08	0.28
C	-0.176	0.839	0.208	-0.85	0.40
D	0.049	1.050	0.146	0.33	0.74
E	0.235	1.266	0.164	1.46	0.14
F	0.214	1.238	0.142	1.51	0.13
G	0.004	1.004	0.185	0.02	0.98
H	0.185	1.204	0.149	1.23	0.22
I	0.020	1.020	0.153	0.13	0.90

Relapse	Estimate	HR	SE	*z*-value	Pr(>|*z*|)
Treatment (Breast-conserving therapy = reference)					
Mastectomy	0.210	1.234	0.083	2.46	**< 0.05**
Age (years)	-0.011	0.989	0.004	-2.88	**< 0.001**
Tumour size in mm	0.042	1.043	0.004	9.61	**< 0.001**
Number of positive lymph nodes	0.282	1.326	0.053	5.34	**< 0.001**
Grade (1 = reference)					
2	0.595	1.813	0.115	5.18	**< 0.001**
3	0.957	2.604	0.132	7.24	**< 0.001**
Multifocality (No = reference)					
Yes	0.342	1.408	0.103	3.23	**< 0.001**

Relapse	Estimate	HR	SE	*z*-value	Pr(>|*z*|)
Histological tumour type (Ductal = reference)					
Lobular	-0.244	0.783	0.139	-1.74	0.08
Mixed	-0.022	0.978	0.191	-0.12	0.91
Other	-0.555	0.574	0.235	-2.32	**< 0.05**
Sublocalisation (Outer quadrants = reference)					
Inner quadrants	0.229	1.258	0.093	2.42	**< 0.05**
Central parts	0.023	1.023	0.145	0.16	0.87
Overlapping lesions	-0.018	0.982	0.091	-0.19	0.85
Lateralisation (Left = reference)					
Right	-0.023	0.977	0.071	-0.32	0.75
Hormonal receptor status (Negative = reference)					
Mixed	-0.112	0.894	0.150	-0.74	0.46
Positive	-0.202	0.817	0.139	-1.40	0.16
HER2 status (Negative = reference)					
Mixed	-0.075	0.928	0.148	-0.50	0.62
Positive	0.243	1.275	0.115	2.05	**< 0.05**
Targeted therapy (No = reference)					
Yes	-0.472	0.623	0.193	-2.39	**< 0.05**
Adjuvant systemic therapy (None = reference)					
Chemotherapy	-0.728	0.483	0.161	-4.42	**< 0.001**
Endocrine therapy	-0.476	0.622	0.115	-4.09	**< 0.001**
Both	-1.011	0.364	0.133	-7.50	**< 0.001**
Auxillary lymph node dissection (No = reference)					
Yes	-0.079	0.924	0.097	-0.81	0.42
Socioeconomic status (Low = reference)					
Medium	0.038	1.038	0.090	0.41	0.68
High	0.149	1.161	0.094	1.57	0.12
Hospital type (General = reference)					
Top-clinical	-0.067	0.936	0.081	-0.81	0.42
Academic	-0.218	0.804	0.154	-1.39	0.16

Relapse	Estimate	HR	SE	*z*-value	Pr(>|*z*|)
Hospital region (A = reference)					
B	0.170	1.185	0.161	1.05	0.29
C	0.079	1.082	0.165	0.48	0.63
D	0.437	1.547	0.124	3.51	**< 0.001**
E	-0.058	0.944	0.155	-0.37	0.71
F	-0.069	0.933	0.139	-0.49	0.62
G	0.084	1.088	0.158	0.53	0.59
H	-0.037	0.964	0.143	-0.26	0.80
I	0.252	1.286	0.129	1.95	0.05

Second primary tumour	Estimate	HR	SE	*z*-value	Pr(>|*z*|)
Treatment (Breast-conserving therapy = reference)					
Mastectomy	-0.012	0.988	0.082	-0.14	0.89
Age (years)	0.026	1.026	0.004	7.23	**< 0.001**
Tumour size in mm	0.000	1.000	0.005	-0.08	0.93
Number of positive lymph nodes	0.117	1.125	0.061	1.94	0.05
Grade (1 = reference)					
2	-0.014	0.986	0.086	-0.16	0.87
3	0.114	1.121	0.116	0.95	0.34
Multifocality (No = reference)					
Yes	0.062	1.064	0.111	0.57	0.57
Histological tumour type (Ductal = reference)					
Lobular	0.151	1.163	0.112	1.34	0.18
Mixed	0.048	1.049	0.174	0.27	0.79
Other	0.125	1.133	0.162	0.76	0.45
Sublocalisation (Outer quadrants = reference)					
Inner quadrants	0.093	1.097	0.090	1.03	0.30
Central parts	0.054	1.056	0.134	0.40	0.69
Overlapping lesions	0.072	1.074	0.087	0.82	0.41
Lateralisation (Left = reference)					
Right	-0.129	0.879	0.069	-1.88	0.06
Hormonal receptor status (Negative = reference)					
Mixed	0.068	1.071	0.157	0.43	0.67
Positive	0.038	1.039	0.144	0.26	0.79

Second primary tumour	Estimate	HR	SE	*z*-value	Pr(>|*z*|)
HER2 status (Negative = reference)					
Mixed	-0.123	0.884	0.145	-0.84	0.40
Positive	-0.160	0.852	0.133	-1.18	0.24
Targeted therapy (No = reference)					
Yes	0.081	1.085	0.224	0.36	0.72
Adjuvant systemic therapy (None = reference)					
Chemotherapy	-0.399	0.671	0.187	-2.09	**< 0.05**
Endocrine therapy	-0.294	0.745	0.107	-2.67	**< 0.01**
Both	-0.550	0.577	0.143	-3.78	**< 0.001**
Auxillary lymph node dissection (No = reference)					
Yes	-0.005	0.995	0.094	-0.06	0.95
Socioeconomic status (Low = reference)					
Medium	-0.032	0.969	0.085	-0.37	0.71
High	0.015	1.015	0.090	0.16	0.87
Hospital type (General = reference)					
Top-clinical	-0.042	0.959	0.080	-0.52	0.60
Academic	0.466	1.594	0.118	3.94	**< 0.001**
Hospital region (A = reference)					
B	-0.055	0.947	0.140	-0.39	0.70
C	-0.257	0.774	0.156	-1.65	0.10
D	-0.302	0.739	0.128	-2.33	**< 0.05**
E	-0.110	0.896	0.136	-0.81	0.42
F	-0.050	0.951	0.123	-0.41	0.69
G	-0.186	0.830	0.153	-1.21	0.23
H	-0.342	0.711	0.135	-2.54	**< 0.05**
I	-0.227	0.797	0.126	-1.81	0.07

References

1. Putter H, Fiocco M, Gekus RB. Tutorial in biostatistics: Competing risk and multi-state models. *Stat Med*. 2007. doi:10.1002/sim.2712

2. Piessens R, de Doncker-Kapenga E, Überhuber CW, Kahaner DK. *Quadpack: A Subroutine Package for Automatic Integration*. Vol 1. Berlin, Heidelberg: Springer Berlin / Heidelberg; 1983.

3. Singletary SE, Greene FL, Sobin LH. Classification of Isolated Tumor Cells: Clarification of the 6th Edition of the American Joint Committee on Cancer Staging Manual. *Cancer*. 2003;98(12):2740-2741. doi:10.1002/cncr.11865

4. Fritz A, Percy C, Jack A, et al. World Health Organization: International Classification of Diseases for Oncology. 2000:240 pages. http://whqlibdoc.who.int/publications/2000/9241545348_eng.pdf.

5. Knol F. Statusontwikkeling van wijken in Nederland 1998-2010. *Soc en Cult Planbur*. 2012.

6. Gass J, Mitchell S, Hanna M. How do breast cancer surgery scars impact survivorship? Findings from a nationwide survey in the United States. *BMC Cancer*. 2019;19(1). doi:10.1186/s12885-019-5553-0
